# Disparities in the Prevalence of Hospitalizations and In-Hospital Mortality Due to Acute Myocardial Infarction Among Patients with Non-Alcoholic Fatty Liver Disease: A Nationwide Retrospective Study

**DOI:** 10.3390/jcm13226946

**Published:** 2024-11-18

**Authors:** Umar Hayat, Faisal Kamal, Muhammad U. Kamal, Wasique Mirza, Tariq A. Ahmad, Manesh K. Gangwani, Dushyant S. Dahiya, Hassam Ali, Shiva F. Naidoo, Sara Humayun, Hayrettin Okut, Muhammad Aziz

**Affiliations:** 1 Department of Internal Medicine, Geisinger Wyoming Valley Medical Center, Wilkes-Barre, PA 18705, USA; wmirza@geisinger.edu (W.M.); tahmad1@geisinger.edu (T.A.A.); shivanaidoo26@gmail.com (S.F.N.); shumayun@geisinger.edu (S.H.); 2 Department of Internal Medicine, Division of Gastroenterology, Thomas Jefferson University, Philadelphia, PA 19107, USA; fkamal36@gmail.com; 3 Essen Healthcare System, Bronx, NY 10458, USA; muhammadumarkamal@gmail.com; 4 Department of Internal Medicine, University of Toledo, Toledo, OH 43614, USA; gangwani.manesh@gmail.com; 5 Department of Gastroenterology and Hepatology, The University of Kansas School of Medicine, Kansas City, KS 66103, USA; dush.dahiya@gmail.com; 6 Department of Gastroenterology, ECU Health Medical Center, Greenville, NC 27858, USA; hassamali155@gmail.com; 7 Department of Population and Public Health, University of Kansas, Wichita, KS 67214, USA; hokut@kumc.edu; 8 Department of Gastroenterology, Bon Secours Mercy Health, Toledo, OH 43608, USA; marajani786@gmail.com

**Keywords:** NAFLD: non-alcoholic liver disease, AMI: acute myocardial infarction, NIS: national inpatient sample, disparities, hospitalizations, in-hospital mortality

## Abstract

***Background*:** Non-alcoholic liver disease (NAFLD) may be associated with cardiovascular diseases; however, only a few studies have analyzed this relationship. We aimed to assess the epidemiologic data and the association between NAFLD and acute myocardial infarction (AMI) in the United States. ***Methods*:** The National Inpatient Sample (NIS) database 2016–2019 was queried using ICD10-CM diagnostic codes to identify hospitalizations of AMI + NAFLD. Essential demographic variables were analyzed to determine the disparities in the prevalence of AMI hospitalizations and deaths among NAFLD patients. Univariate and multivariate logistic regression models determined the association between NAFLD and AMI hospitalizations and death. ***Results:*** Among the total 5450 NAFLD patients hospitalized with AMI, 5.11% (279) died. Females were less likely to be admitted and die due to AMI than males. Younger patients (<50) were less likely to be hospitalized and die than those ≥50. Compared to the white population, black patients were less likely; however, Hispanics, Asians, and Pacific Islanders were more likely to be hospitalized. Race was not found to affect hospital mortality. On multivariate analysis, NAFLD was associated with higher odds of AMI hospitalization [OR 1.55, 95% CI 1.51–1.60, *p* < 0.01] and death [OR 1.96, 95% CI 1.74–2.21, *p* < 0.01]. ***Conclusions*:** Older white males with NAFLD had a higher prevalence of AMI hospitalizations and mortality.

## 1. Introduction

Non-alcoholic fatty liver disease (NAFLD) is the most common form of liver disease in the United States and worldwide, with an estimated global prevalence of ~25% and constantly escalating [1,2,3]. It is characterized by excess fat accumulation in the liver parenchyma without excessive alcohol intake. NAFLD is categorized into several clinicopathologic forms ranging from simple fatty liver with no or minimal inflammation to severe Non-alcoholic steatohepatitis (NASH) condition, which causes liver fibrosis leading to cirrhosis and hepatocellular carcinoma [4,5]. Among NAFLD patients, about 3–5% meet the criteria of NASH [1,3]. Weight loss has been considered the only treatment option for NAFLD. More recently, some natural products have shown promising results [6,7]. These products regulate lipid metabolism and improve oxidative stress, thus alleviating liver inflammation, mostly in animal models [6,7].

NAFLD is associated with higher odds of liver-related mortality and morbidity, but its impact on acute cardiovascular disease (CVD) events is still debatable. CVD is quite prevalent in NAFLD patients [2], contributing to at least 40% of total deaths in NAFLD and thus constitutes the leading cause of death [2,8]. Studies have reported different mechanisms that can contribute to coronary events in patients with NAFLD, like deranged glucose and lipid metabolism, insulin resistance, hypercoagulable states, excessive oxidate damage, and established cardiovascular (CV) risk factors [9]. CVD in NAFLD is also exhibited by subclinical atherosclerosis, subclinical acute myocardial infarction (AMI), carotid atherosclerotic plaques, and higher severity of coronary artery disease in the NAFLD population [10,11]. Two recent meta-analyses have reported higher odds of acute cardiovascular events (myocardial infarction and stroke) in NAFLD patients, with a higher risk in NASH patients [12,13]. In a German primary care setting study, authors concluded that NAFLD constitutes an independent risk factor for coronary heart disease (CHD), AMI (acute myocardial infarction), and atrial fibrillation [14]. Some cohort studies have also reported an association between NAFLD and AMI. However, they only included patients with ST-segment elevation myocardial infarction, acute coronary syndromes, or those undergoing coronary angiography [15,16,17,18]. Therefore, the interpretation of their findings is limited by selection bias.

Due to these controversial findings, it is unclear if NAFLD plays a role in developing acute myocardial infarction as an independent risk factor. Moreover, how gender, age, and race affect morbidity and in-hospital mortality among NAFLD patients due to AMI needs further attention. To explore the data ambiguity surrounding this question, we examined the association between NAFLD and AMI in the United States population using the National Inpatient Sample (NIS) database. We also examined the racial disparities in hospitalizations and in-hospital mortality due to AMI among NAFLD patients.

## 2. Materials and Methods

### 2.1. Data Source

The NIS is one of the largest publicly available, nationally representative all-payer inpatient care databases in the United States. It is one of the database projects of the Healthcare Cost and Utilization Project (H-CUP) developed by the Agency of Healthcare Research and Quality (AHRQ) to report information on patient hospitalizations and discharges from over 1000 hospitals in 45 states [19]. Different geographic regions randomly stratify this data to represent over 20% of US hospitalizations to protect the privacy of patients, hospitals, and physicians. It incorporates clinical and healthcare resource utilization information of approximately 8 million annual hospitalizations. Since NIS is a deidentified national database, patient consent or institutional board review (IRB) is not required for data utilization. However, we completed the data use agreement course to access the database and obtained an agreement code before data use.

### 2.2. Study Design and Patient Population

**Figure 1:**
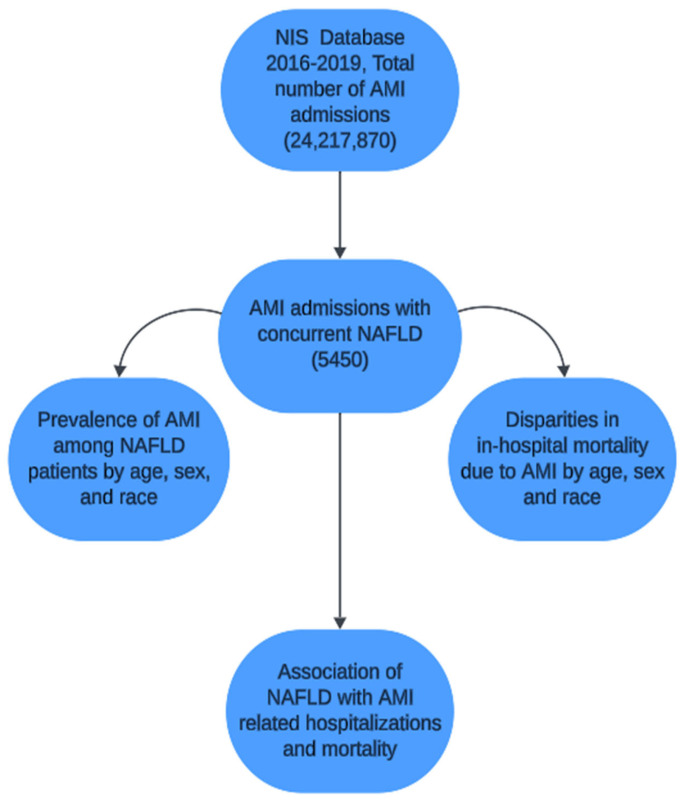
Methods and materials flow chart.

The National Inpatient Sample (NIS) database 2016–2019 was queried to identify the population of our interest using (ICD10-CM) codes to identify a cohort of inpatient admissions with a primary diagnosis of AMI and secondary concurrent diagnosis of NAFLD. We reviewed all previously used and validated diagnostic codes to accurately categorize patients with AMI and NAFLD [20,21,22]. Two comparative groups of patients were created from the final patient population cohort. (1) the patients admitted with AMI and had a concurrent diagnosis of NAFLD (2) those admitted with AMI but did not have NAFLD. Essential demographic variables were analyzed to determine the disparities in the prevalence of AMI among NAFLD patients by sex, age, and race. All patients > 18 years of age were included in the study. To compare the prevalence of AMI among patients with NAFLD, we further stratified patients into two age groups: >18 to 50 and ≥50. We compared the demographic, hospital (region, size, and teaching status), and patient-level (income status and insurance) characteristics. Moreover, the individual comorbidities were assessed between the NAFLD + AMI and AMI-only groups. The flow chart of materials and methods is shown below (Figure 1).

### 2.3. Study Outcomes

The primary outcome of this study was to determine the disparities in the prevalence of AMI among the patients who had NAFLD by sex, age, and race. The secondary outcomes were to determine the disparities in in-hospital mortality due to AMI among NAFLD patients by sex, age, and race and the association of NAFLD with AMI hospitalizations and mortality.

### 2.4. Covariable and Comorbidities

Many co-variables were included for a robust analysis to minimize the number of confounders. NIS database included most of the covariables such as age, race, gender, insurance type, hospital type, teaching status of the hospital, hospital bed size and location (urban, urban teaching, rural), and region of the data in the USA. Comorbidities included in the analysis were hypertension, diabetes mellitus, hyperlipidemia, chronic kidney disease, and congestive heart failure, which were included independently using ICD-10 codes (Supplementary Materials, ICD-10 codes for the variables).

### 2.5. Statistical Analysis

All the statistical analyses were performed using SAS software, version 9.4 (Austin, TX, USA). For categorical variables, descriptive statistics were presented as frequencies with percentages and for continuous variables as means with standard deviations. Baseline characteristics were compared using independent samples *t*-test for continuous variables, Pearson χ2 test, and Fisher exact test for categorical variables. A univariate logistic regression model using demographic characteristics was used to estimate odds ratios with 95% confidence intervals to determine the likelihood of having AMI and in-hospital mortality due to AMI among NAFLD patients by sex, age, and race. Multivariate logistic regression analysis was used to analyze the association of NAFLD and AMI and in-hospital mortality due to AMI after adjusting for all the potential confounders. The threshold for statistical significance was defined as *p*-value < 0.05.

## 3. Results

Between 2016 and 2019, a total of 24,217,870 patients admitted to the hospital with a primary diagnosis of AMI were identified. Of those, 5450 patients had a concurrent diagnosis of non-alcoholic fatty liver disease. Among them, 3351 (57.56%) were males, and 2099 (42.41%) were females. 4487 (82%) patients were ≥50 years of age, and 963 (18%) patients were <50 years of age. 3709 (68.05%) were white patients, while 1741 (31.95%) were non-white patients. Among non-whites, 467 (8%) were Black, 710 (13%) were Hispanic, 199 (3%) were Asian or Pacific Islander, 54 (0.6%) were Native Americans, and 311 (5%) were others (Table 1). The AMI + NAFLD group had a higher prevalence of comorbidities than the AMI + no NAFLD group.

### 3.1. The Association of NAFLD with Hospitalization Due to AMI by Gender, Age, and Race

Compared to the males, females were less likely to be hospitalized in the AMI + NAFLD group compared to the AMI + no NAFLD group [OR 0.46, 95% CI 0.43–0.48, *p* < 0.01]. Also, younger patients (<50) with NAFLD were less likely to be admitted to the hospital due to AMI compared to the older patients (≥50) [OR 0.41, 95% CI 0.38–0.44, *p* < 0.01]. Moreover, compared to the white population, blacks [OR 0.57, 95% CI 0.51–0.61, *p* < 0.01] were less likely to be admitted to a hospital with AMI. However, Hispanics [OR 1.16, 95% CI 1.07–1.25, *p* < 0.01] and Asian and Pacific Islanders [OR 1.31, 95% CI 1.13–1.51, *p* < 0.01] were more likely to be admitted to the hospital with AMI than whites (Table 1). On multivariate analysis, the NAFLD was associated with a higher odd of hospitalizations due to AMI [OR _adjusted_ 1.55, 95% CI 1.51–1.60, *p* < 0.01] after adjusting for age, race, sex, patient’s smoking status, BMI, insurance status, hospital location, hospital teaching status, history of previous AMI, congestive heart failure, diabetes mellitus, chronic kidney disease, hyperlipidemia, and hypertension (Table 2).

### 3.2. The Association of NAFLD with In-Hospital Mortality Due to AMI by Gender, Age, and Race

279 (5.11%) patients died in the hospital. Males with NALFD admitted to the hospital due to AMI were likelier to die than females [OR 1.58, 95% CI 1.25–1.91, *p* < 0.01]. Also, patients ≥ 50 years had higher odds of death due to AMI if they had NAFLD compared to those < 50 years [OR 4.29, 95% CI 2.94–6.27, *p* < 0.01]. Compared with the white population, there was statistically no significant difference in the in-hospital mortality of black patients [OR 0.79, 95% CI 0.54–1.15, *p* = 0.02], Hispanics [OR 1.21, 95% CI 0.85–1.74, *p* = 0.03], Asian and Pacific Islanders [OR 0.95, 95% CI 0.45–2.03, *p* = 0.01], and Native Americans [OR 0.58, 95% CI 0.08–4.17, *p* = 0.01]. On multivariate analysis, the NAFLD patients had higher odds of in-hospital mortality if admitted to the hospital with AMI [OR _adjusted_ 1.96, 95% CI 1.74–2.21, *p* < 0.01] after controlling for age, race, sex, patient’s smoking status, BMI, insurance status, hospital location, hospital teaching status, history of previous AMI, congestive heart failure, diabetes mellitus, chronic kidney disease, hyperlipidemia, and hypertension (Table 2).

## 4. Discussion

In this large population-based study using the NIS database, we reported the disparities in hospitalization among NAFLD patients due to AMI by age, gender, and race. We also reported the disparities in in-hospital mortality due to AMI among NAFLD patients. Elderly patients with NAFLD had a higher likelihood of hospitalizations and in-hospital mortality due to AMI than younger patients. Male patients were likelier to have AMI hospitalizations and in-hospital mortality than females. Also, the white population was more likely to be admitted to the hospital due to AMI if they had NAFLD. NAFLD was associated with an increased risk of hospitalization and in-hospital mortality among the patients admitted with AMI. Recent studies suggested a relationship between NAFLD and cardiovascular disease (CVD) beyond the common risk factors for both conditions [23,24]. Furthermore, both diseases reciprocate in terms of etiology and severity, and individuals with CVD should be screened for NAFLD [25,26]. In a recent population-based study, Ghoneim et al. demonstrated the prevalence of AMI among NASH patients and found that NASH has a strong association with AMI [21]. The pathophysiological mechanism of how NAFLD is associated with an increased risk of CVD involves several complex mechanisms. The liver plays a crucial role in glucose and lipid metabolism. A liver injury resulting from NAFLD is associated with a deranged lipid profile, an independent risk factor for CVD [27]. In NAFLD, there is an imbalance between the lipid acquisition and lipid disposal by the liver, which may precede the following cascade of inadequate uptake of circulating lipids by the liver, abnormally enhanced fatty acid oxidation leading to increased production of oxygen free radicals, increased de-novo lipogenesis (DNL) by the liver, and an altered export of lipids and very low-density lipoproteins (VLDL) [28]. This cascade may further precede the abnormal fat accumulation in the liver and cause significant triglyceride-rich VLDL mobilization to the peripheral tissues [29]. These VLDL particles facilitate the transport of liver fat to the peripheral tissue and initiate a clump of plasma lipoprotein abnormalities and atherogenic dyslipidemia characterized by high serum cholesterol and triglycerides level, low high-density lipoproteins (HDL). As a result, many atherogenic lipoprotein phenotypes, such as small dense low-density lipoprotein (LDL) particles and triglyceride-rich lipoproteins and their remnants, intermediate-density lipoprotein (IDL), are generated. The increased production of this apolipoprotein-B containing lipoproteins in the serum serves as a substrate for atherosclerosis development, eventually leading to CVD [30,31]. Furthermore, NAFLD and AMI are the end-organ damage manifestations of the systematic metabolic syndrome (MeS), which explains NAFLD’s contribution to the increased risk of CVD [32].

We found a higher prevalence of AMI among NAFLD patients. Haddad et al., in a meta-analysis of six studies with 25,837 patients, reported that the NAFLD patients had a significantly higher risk of clinical coronary artery disease (AMI) compared to those without NAFLD (RR 2.26; 95% CI 1.04–4.92; *p* < 0.001) [33]. Another study reported NASH as an independent risk factor for an increased risk of acute coronary events [34]. Additionally, our findings reveal that the risk of AMI was higher among males with NAFLD than their female counterparts across all the age groups. Studies have shown that females with AMI are younger and more likely to have a higher proportion of comorbidities than their male counterparts [35]. Also, when stratified by age, we found a higher prevalence of AMI among older patients (≥50) with NAFLD, suggesting an increased relative risk of CVD in this population. Age correlates with NAFLD duration as well as CVD. With increasing age, the more advanced liver disease might have been attributed to severe atherogenic dyslipidemia, eventually leading to CVD. Ghoneim et al. also observed a relative increase in the absolute risk of AMI with increasing age among NAFLD patients [21]. Furthermore, NAFLD patients were likelier to die if admitted to the hospital with AMI. In a study of 360 NAFLD patients admitted to the hospital with STEMI, Keskin et al. observed an increase in hospital and 3-year mortality compared to those who did not have NAFLD [17].

Racial differences in the incidence and prevalence of AMI exist across racial and ethnic groups; however, there has been an overall decrease in the incidence and prevalence of AMI across all racial and ethnic groups over time [36,37]. Recent studies have demonstrated that black patients were more prone to be hospitalized with AMI than white patients [38,39,40]. We found that NAFLD was more prevalent among white patients than non-white patients, and white patients with NAFLD were more prone to AMI. Compared to the non-white population, white patients had more comorbidities that might have contributed to the CVD and the deranged lipid profile due to NAFLD. Socioeconomic status also determines hospital admission due to AMI, as black patients with Medicare beneficiaries are less likely to be admitted than white patients [41]. Similarly, white patients with NAFLD were more prone to die if admitted to the hospital due to AMI than non-white patients. Young et al. found that black patients presenting with STEMI had the lowest in-hospital mortality rates despite lower rates of timely angiography and lower use of drug-eluting stents (DES) [42]. They found higher in-hospital mortality among Asian patients compared to white patients. Moreover, a recent meta-analysis evidenced that NAFLD increases the risk of AMI, and several randomized controlled trials showed that there is a significant decrease in cardiovascular events through the treatment of non-alcoholic fatty liver disease [43,44].

Our study findings must be interpreted by considering the following limitations. First, it is an observational study using the NIS database and does not address the causation effect of NAFLD on morbidity and mortality due to AMI. The NIS database collects data for billing purposes from approximately 20% of all US hospitals, and findings from this database cannot be generalized to the whole population. Also, these findings can be limited by the erroneous coding despite using all the ICD-10 codes, which are associated with higher specificity and positive predictive value for diagnosis for our outcomes of interest. Although the NIS database provides a detailed assessment of in-hospital patient outcomes, it does not provide any information on baseline laboratory and imaging data, readmission of patients with similar complaints, and mortality outside the hospital. Therefore, a selection bias of NAFLD patients who had AMI cannot be entirely excluded from our analysis. Lastly, there is a limitation to checking the validity of the diagnosis of AMI from the used ICD-10 codes because all the patients are de-identified in the database, and it was not possible to segregate patients who had AMI due to either coronary artery disease or demand-related ischemia (Type II MI). Acknowledging this issue, we used all the validated codes known for AMI, which the previously published studies used. Finally, sarcopenia could be related to a higher risk of cardiovascular mortality [45]. Although a recent meta-analysis did not find this association, it could be considered a confounder for AMI-related mortality, especially among elderly patients [46]. However, we could not include this variable due to unavailability in the database. Our study has several strengths: (1) The most extensive population-based study has ascertained the racial disparities in the prevalence of AMI and in-hospital mortality among NAFLD patients. (2) We provided thorough epidemiological information on the risk of AMI among NAFLD patients and discussed all the possible risk factors. (3) We used the largest and most inclusive national inpatient sample database in the USA, which enhances the generalizability and external validity of inferred study findings. (4) The large sample size from the NIS database provided an excellent source for further evaluation of risk factors causing disparities in the prevalence and outcomes of AMI among NAFLD patients.

## 5. Conclusions

In conclusion, among NAFLD patients, there are noteworthy disparities in hospitalizations and in-hospital mortality due to AMI by age, gender, and race. Moreover, a significant association was found between NAFLD and AMI. Risk stratification must be performed among NAFLD patients for early detection and aggressive risk factor modification to prevent cardiovascular diseases. Prospective cohort studies need to be conducted to unravel this association further.

## Figures and Tables

**Table 1:** (**a**) Baseline characteristics of the subjects (**b**) unadjusted and adjusted odds ratio of having an associated diagnosis of AMI and NAFLD (**c**) unadjusted and adjusted odds ratio of in-hospital mortality among NAFLD patients due to AMI (**d**) multivariate analysis for NAFLD and AMI association.

Variables	AMI Without NAFLD (n = 24,212,420)	AMI with NAFLD (n = 5450)	*p*-Value
**(a) Baseline Patient and Hospital Characteristics**			
Age (%)	>50 yr. 15,959,913 (65.90) <50 yr. 8,257,626 (34.09)	>50 yr. 4487 (82%) <50 yr. 963 (18%)	*p* < 0.01
Female (%)	13,940,284 (57.57)	2099 (42.41%)	*p* < 0.01
Race (%)			
White	15,787,986 (67.30)	3709 (68.05)	*p* = 0.04
Hospital Type (%)			
Urban	2,192,821 (9.05)	292 (2.0)	*p* < 0.01
Teaching	16,670,307 (68.83)	3956 (72.6)	*p* < 0.01
Insurance Type (%)			
Medicare		2542 (47.65)	
**Comorbidities (%)**			
History of previous MI	1,337,360 (5.52)	805 (14.77)	*p* < 0.01
Obesity	3,800,115 (15.7)	1938 (35.55)	*p* < 0.01
Congestive heart failure	3,645,424 (15.05)	1632 (29.94)	*p* < 0.01
Chronic Kidney Disease	3,257,055 (13.45)	1056 (19.37)	*p* < 0.01
Peripheral Vascular disease	675,227 (2.7)	229 (4.2)	*p* < 0.01
Diabetes Mellitus	2,701,175 (11.15)	906 (16.62)	*p* < 0.01
Hypertension	8,178,454 (33.77)	2356 (43.22)	*p* < 0.01
Hyperlipidemia	6,745,123 (27.85)	2986 (54.78)	*p* < 0.01
Smoking	5,246,520 (21.66)	1487 (27.28)	*p* < 0.01
**(b) Logistic regression for hospitalizations due to AMI**			
**Variables**	**Odds Ratio**	**95% CI**	***p* < 0.01**
Sex			
Male (reference)	-	-	-
Female	0.46	0.43–0.48	*p* < 0.01
Age			
>50 (reference)	-	-	-
<50	0.41	0.38–0.44	*p* < 0.01
Race			
White (reference)	-	-	
Black	0.57	0.51–0.61	*p* < 0.01
Hispanic	1.16	1.07–1.25	*p* < 0.01
Asian and Pacific Islanders	1.31	1.13–1.51	*p* < 0.01
NAFLD and AMI (* Adjusted)	**1.55**	**1.51–1.60**	***p* < 0.01**
**(c) Logistic regression analysis for in-hospital mortality**			
**Variables**	**Odds Ratio**	**95% CI**	***p*-value**
Sex			
Female (reference)	-	-	-
Male	1.58	1.25–1.91	*p* < 0.01
Age			
<50 (reference)	-	-	-
>50	4.29	2.94–6.27	*p* < 0.01
Race			
White (reference)	-	-	
Black	0.79	0.54–1.15	*p* = 0.02
Hispanic	1.21	0.85–1.74	*p* = 0.03
Asian and Pacific Islanders	0.95	0.45–2.03	*p* = 0.01
Native Americans	0.58	0.08–4.17	*p* = 0.01
**(d) Multivariate Analysis for NAFLD and AMI association**			
NAFLD and AMI (* Adjusted)	**1.96**	**1.74–2.21**	***p* < 0.01**

NAFLD: Nonalcoholic fatty liver disease, AMI: acute myocardial infarction. * Adjusted for age, race, sex, patient’s smoking status, BMI, insurance status, hospital location, hospital teaching status, history of previous AMI, congestive heart failure, diabetes mellitus, chronic kidney disease, hyperlipidemia, and hypertension.

**Table 2:** Adjusted odds ratio having an associated diagnosis of acute myocardial infarction with NAFLD based on subject co-morbidities.

Odds Ratio Estimates			
Effect	Point Estimate	95% Confidence Limits	
NAFLD No NAFLD vs. NAFLD	1.556	1.514	1.600
AGE_GROUP ≥50 vs. <50	2.641	2.616	2.666
RACE Black vs. white	0.827	0.821	0.833
Hispanic vs. White	0.947	0.939	0.955
Asian or Pacific Islander vs. White	1.187	1.170	1.204
Native American vs. White	1.100	1.066	1.135
Other race vs. White	1.091	1.075	1.107
FEMALE Male vs. Female	1.618	1.610	1.626
Hyperlipidemia No Hyperlipidemia vs. Hyperlipidemia	0.513	0.511	0.516
Smoking No smoking vs. smoking	1.072	1.066	1.078
Insurance Medicaid vs. Medicare	0.960	0.952	0.969
Private Insurance vs. Medicare	1.235	1.227	1.243
Self-pay vs. Medicare	1.763	1.740	1.785
No charge vs. Medicare	1.689	1.622	1.758
Other vs. Medicare	1.116	1.099	1.133
Hospital teaching status Teaching vs. Non-teaching	1.119	1.108	1.130
Diabetes 1 and 2 Non-Diabetic vs. Diabetic	0.907	0.901	0.913
PVD No PVD vs. PVD	0.744	0.737	0.752
CKD No CKD vs. CKD	0.853	0.847	0.859
CHF No CHF vs. CHF	0.457	0.454	0.460
Previous MI No MI vs. MI	0.575	0.571	0.579
Hypertension No Hypertension vs. Hypertension	0.840	0.835	0.846

Abbreviations and codes: CKD; Chronic kidney disease, CHF; Congestive heart failure, PVD; Peripheral vascular disease, MI; Myocardial Infarction.

## Data Availability

3rd party data: No additional data are available to share. Restrictions apply to the availability of these data. Data were obtained from the Healthcare Cost and Utilization Project (H-CUP) and are available from HCUP-US Databases (https://www.ahrq.gov) with permission.

## Supplementary Materials

The following supporting information can be downloaded at: https://www.mdpi.com/article/10.3390/jcm13226946/s1, Table S1: ICD-10 codes for all the study variables.
