# Supplementary material for: Disparities in the Prevalence of Hospitalizations and In-Hospital Mortality Due to Acute Myocardial Infarction Among Patients with Non-Alcoholic Fatty Liver Disease: A Nationwide Retrospective Study
Source: J Clin Med. 2024 Nov 18;13(22):6946. doi: 10.3390/jcm13226946 (PMC11595205; doi:10.3390/jcm13226946)
Supplement: Supplementary file 1 [file jcm-13-06946-s001.zip › jcm-3277721-supplementary.pdf]

Supplementary Table S1:

A Supplementary file, including the ICD-10 codes used for all the study variables, has been submitted.

Supplementary material

Supplementary Table S1. ICD-10 codes for all the study variables.

	ICD-10 codes
Diagnosis codes	
AMI	I21, I21.0, I21.01, I21.02, I21.09, I21.1<br>I21.11, I21.19, I21.2, I21.3, I21.4
History of previous MI	I252
NAFLD	K760
Hyperlipidemia	E785
Obesity	E660, E6601, E6609, E661, E662, E668, E669
Congestive heart failure	I50, I502, I5020, I5021, I5022, I5023, I503, I5030, I5031, I5032, I5033, I504, I509
Comorbidities	
Chronic kidney disease	N18, N181, N182, N183, N184, N185, N189
Old MI	I252
Old PCI	Z9861
Old CABG	Z951
Old pacemaker	Z950
Atrial fibrillation/flutter	I48
Chronic obstructive pulmonary disease	J41, J42, J43, J44

Carotid artery disease	I652
Old stroke	I63
Hypertension	I10
Peripheral vascular disease	I739
Hypothyroidism	E03
Diabetes mellitus type 1 & 2	E10, E11
Obesity	E660, E6601, E6609, E661, E662, E668, E669
Congestive heart failure	I50
Chronic kidney disease	N18
Liver disease	K70, K71, K72, K73, K74, K75, K76, K77
Electrolyte derangement	E870, E871, E872, E873, E874, E875, E876
Maintenance dialysis	Z992
Oxygen dependence	Z9981
Smoking	Z87891, F17200
Anemia	D50, D51, D52, D53, D55, D56, D57, D58, D59, D60, D61, D62, D63, D64
